# Right Ventricular Functionality Following Hemodialysis Initiation in End-Stage Kidney Disease—A Single-Center, Prospective, Cohort Study

**DOI:** 10.3390/medicina57070704

**Published:** 2021-07-10

**Authors:** Ana Tanasa, Alexandru Burlacu, Iolanda Valentina Popa, Adrian Covic

**Affiliations:** 1 Nephrology Clinic, Dialysis, Renal Transplant Center—‘C.I. Parhon’ University Hospital, 700503 Iasi, Romania; tanasa.ana@umfiasi.ro (A.T.); adrian.covic@umfiasi.ro (A.C.); 2 Faculty of Medicine, University of Medicine and Pharmacy “Grigore T Popa”, 700115 Iasi, Romania; 3 Institute of Cardiovascular Diseases “Prof. Dr. George I.M. Georgescu”, 700503 Iasi, Romania

**Keywords:** ESKD, hemodialysis, right ventricle, 2D-speckle tracking echocardiography, strain, mortality

## Abstract

*Background and Objectives:* Two-dimensional speckle tracking echocardiography (2D-STE) is viewed as an outstanding technique, competent of uncovering earlier subclinical myocardial anomalies compared to conventional echocardiography. A few endeavors adopted 2D-STE as a tool to estimate right ventricular (RV) function in subjects with end-stage kidney disease (ESKD). There is no published prospective study on an adult ESKD cohort exploring the consequences of commencing elective hemodialysis (HD) on RV behavior. *Materials and Methods*: We investigated the RV systolic function using traditional (tricuspid annular plane systolic excursion—TAPSE, RV fractional area change—FAC) and 2D-STE (RV free wall longitudinal strain—RVFWLS) parameters following the initiation of HD. We enrolled 79 consecutive patients with ESKD and assessed them in four steps—at baseline, before HD, and at 3, 6, and 12 months. *Results*: RVFWLS, FAC, and TAPSE values had a significant increase at 3, 6, and 12 months from baseline (*p* < 0.001) and a significant increase at 6 months from 3 months (*p* < 0.001). However, differences between 12 months and 6 months were not significant (*p* > 0.05) according to Dunn–Bonferroni post hoc tests. Seventeen deaths were recorded before the completion of the study. RVFWLS, FAC, and TAPSE values significantly decreased at 3 and 6 months in all 17 deceased patients, in clear opposition with the values survivors had. All the studied parameters had a significant prediction power on mortality (*p* < 0.001) having an outstanding performance: baseline-RVFWLS (AUC: 1.000 (95% C.I.: 1.000–1.000)), baseline-FAC (AUC: 0.974 (95% C.I.: 0.942–1.000)), and baseline-TAPSE (AUC: 0.962 (95% C.I.: 0.920–1.000). *Conclusions*: Our study is the first to investigate RV function by 2D-STE and correlate it with traditional methods in patients with ESKD before and after the initiation of HD. RV function was significantly ameliorated at 3, 6, and 12 months compared to the pre-HD values. FAC and RVFWLS gain an outstanding prognostic role on mortality in this population.

## 1. Introduction

Cardiovascular disease (CVD) continues to be the most important cause of major ad-verse cardiovascular events (MACE) and death in end-stage kidney disease (ESKD) patients, as reported by the international registries [1]. The pathogenesis of CVD in this population is complex, being related to traditional, known cardiovascular (CV) factors, but also to uremia-specific processes (e.g., alterations in phosphate and calcium metabolism, overt anemia, arterial remodeling by protein carbamylation, and hemodynamic overload) [2].

A distinct and distressing moment in the life of an ESKD patient is the initiation of hemodialysis (HD) sessions [3]. The patient becomes reliable upon medical technology, this matter delivering derogatory psychological and even pathophysiological reactions. Relatively few studies have dipped into detailed CV changes coming after the initiation of dialysis.

Conventional echocardiography may not be capable enough to expose precocious cardiac abnormalities in CKD patients [4], but two-dimensional speckle tracking echocardiography (2D-STE) is viewed as an outstanding technique in the recent past competent of uncovering subclinical myocardial anomalies in the left ventricle (LV) even when the LV ejection fraction (LVEF) is average [5,6].

A few investigations also adopted 2D-STE as a tool to appraise the right ventricular (RV) functionality in subjects with ESKD [7]. To our knowledge, there is no published prospective study on an adult ESKD cohort exploring the effects on RV after commencing elective HD. This study aimed to interrogate the transitions of RV function and mechanics prospectively, using both traditional and 2D-STE parameters, following the initiation of HD in ESKD stable subjects.

## 2. Materials and Methods

### 2.1. Study Population

Our research enrolled 79 consecutive patients with ESKD starting HD and assessed in 4 steps—at baseline, before HD, and at 3, 6, and 12 months. The current investigation was conducted between December 2019 to December 2020 at the “Dr. C. I. Parhon” Hospital, Department of Nephrology, Iași, Romania.

The participants were clinically stable, in sinus rhythm, without any acute diseases, and with a proper acoustic window for echocardiography and strain analysis.

The exclusion criteria were: unwillingness to participate in the study, indication for acute HD, severe pulmonary hypertension and other severe valvular diseases, arrhythmias (including atrial fibrillation), disabling stroke, active malignancy, and LV dysfunction or any other comorbidities which may limit the lifespan of the subject to less than 12 months.

Of 79 patients, only 62 of them finished the study at 12 months, with 17 deaths recorded from CV causes.

### 2.2. Study Design

This investigation was a prospective cohort study. The local Ethical Commission approved the design of the research. Patients who met the eligibility criteria were recorded after studying principles and signing the informed consent. There was no interference to correct the subjects’ routines regarding their medication or water drinking constraints for the duration of the study. Patients started HD with central venous catheters and transitioned to arteriovenous fistula access within 90 days. HD sessions were programmed as three and a half hours.

All the recruited subjects received periodical clinical and echocardiography evaluation at 3, 6, and 12 months. Our main goal was to determine if HD could have favorable aftermath on the function and mechanics of the RV, including subclinical impairment assessed by 2D-STE. In addition, any evaluated parameter was assessed for any potential prognostic role.

### 2.3. Transthoracic Echocardiography

All echocardiographic evaluations were achieved with commercial ultrasound systems (General Electric, Vivid 95), and strain analysis was performed with customized computer software (EchoPAC Version BT13, GE Healthcare).

We obtained the apical 4-chamber view focused on the RV (with patient in left lateral decubitus position) to calculate distinct indicators of RV size and function, and then the offline analysis of the recorded images was done using computer. Additional dedicated images optimizing the free wall right ventricle strain were acquired at high frame rates (>70 frames/s). All images were stored digitally, and analysis was performed offline.

Only cine loops with good quality images acquired at high frame rates were utilized.

A first echocardiographic evaluation was made within 24 h before the first HD session, and all the indices were also investigated at 3, 6, and 12 months. A professional ultrasonographer gathered all the data using the standard protocol. In order to ensure an unbiased outcome, two other experienced ultrasonographers (blinded to the clinical settings of the subjects) analyzed and interpreted the offline images independently.

Although all patients received a comprehensive evaluation of the size and function of all cardiac chambers, in this study, we reported only those concerning the RV systolic function and mechanics.

Firstly, we focused on the traditional evaluation of the systolic function using M-mode measurements for tricuspid annular plane systolic excursion (TAPSE) and two-dimensional standard echocardiography for fractional area change (FAC) using an RV-focused apical four-chamber view. TAPSE represents an estimation of RV longitudinal function, having satisfactory correspondence with other parameters measuring RV overall systolic function, with a value of less than 17mm being deeply evocative for RV systolic dysfunction. TAPSE is defined by the distance of systolic excursion of the RV annular segment along its longitudinal plane from a standard apical 4-chamber window [8]. FAC, determined with 2D echocardiography in both systole and diastole, is another parameter that offers a good perception of global RV systolic function; if the tracing is carefully done to include the apex, free wall, or trabeculae in the RV chamber, a value of less than 35% implies RV systolic decay [9]. FAC is defined as (end-diastolic area − end-systolic area)/end-diastolic area × 100.

### 2.4. Two-Dimensional Speckle Tracking Echocardiography

The strain appraisal was made on an offline groundwork. We manually traced the RV endocardium at the end of the ventricular diastole by selecting 3 or 6 points of interest (usually basal, medial, and apex); the software automatically drew the desired contour on adjacent images. Region of interest (ROI) was estimated, manually tailored to fit the thickness of the RV free wall as well as of the interventricular septum. Subsequently, the specific deformation curves were displayed, with automatic calculation of the global RV strain and the free wall (RVFWLS). FAC was defined as (end-diastolic area − end-systolic area)/end-diastolic area × 100. RVFWLS was defined as the mean longitudinal peak systolic strain of three segments of the RV free wall, according to the established guidelines [8]. Our study used the prospective analysis of only the free RV wall strain, according to international recommendations, to avoid interfering with the LV systole. Although an international agreement for normal values is currently deficient, we considered a value of RV free wall strain below 19% to be pathological, as suggested by large, recently published multicenter studies [10].

We averaged the two measurements offered by our ultrasonographers for each echocardiographic evaluation considering the interobserver variability for RV strain.

### 2.5. Statistical Analysis

All the data from the study were analyzed using IBM SPSS Statistics 25. Quantitative variables were tested for normal distribution using the Shapiro–Wilk test and were written as averages with standard deviations or medians with interquartile ranges. Qualitative variables were written as counts or percentages. Quantitative independent variables were tested using the Mann–Whitney U test, according to their distribution, while quantitative paired variables were tested using Friedman’s tests with Dunn–Bonferroni post hoc tests. Qualitative variables were tested using Fisher’s Exact tests. ROC curves were used for establishing cutoff values in the prediction of mortality.

## 3. Results

### 3.1. Baseline Characteristics

This investigation was performed on 79 consecutive hypertensive subjects with ESRD, including 44 men and 35 women who started hemodialysis and followed for 12 months, with 62 patients completing the study and 17 death were recorded at the end of it.

Data from Table 1 show the characteristics of the analyzed patients. For the categorical variables that denote the presence or absence of a clinical condition (e.g., obesity, smoking), Table 1 illustrates the absolute and relative number of patients with that condition (denoted by the “+” sign) and the absolute and relative number of patients in which that condition is absent (denoted by the “−” sign).

The average value of hemoglobin was 9.75 ± 1.77 g/dL with a median of 9.7 g/dL, with 36.7% of the patients having anemia. The average value of uric acid was 7.46 ± 1.92 mg/dL with a median of 7.25 mg/dL, with 40% of the patients having hyperuricemia. The average value of the volumetric ejection fraction was 54.35 ± 9.74% with a median value of 55%, with 6.3% of the patients having a value of less than 40% EF%. Of the analyzed patients, 21.5% died before the 12 months check-up.

Among the analyzed parameters, we observed that patients that died were significantly older (median = 70 years vs. 60 years, *p* = 0.004), had a significantly lower EF% (median = 53% vs. 56.5%, *p* = 0.024), were significantly more associated with diabetes (58.8% of the dead patients with DM vs. 25.8% of the survivors with DM, *p* = 0.018), and were more associated with NYHA Class III heart failure (17.6% of the dead patients with NYHA III vs. 0% of the survivors with NYHA III, *p* = 0.015). Other parameters were not significantly different in comparison with mortality (*p* > 0.05).

### 3.2. Echocardiographic Variables and Their Prospective Dynamics

TAPSE, FAC, and strain values assessed with 2D-STE at baseline (before the initiation of the first session of HD) and 3, 6, and 12 months are depicted in Table 2.

**Figure 1:**
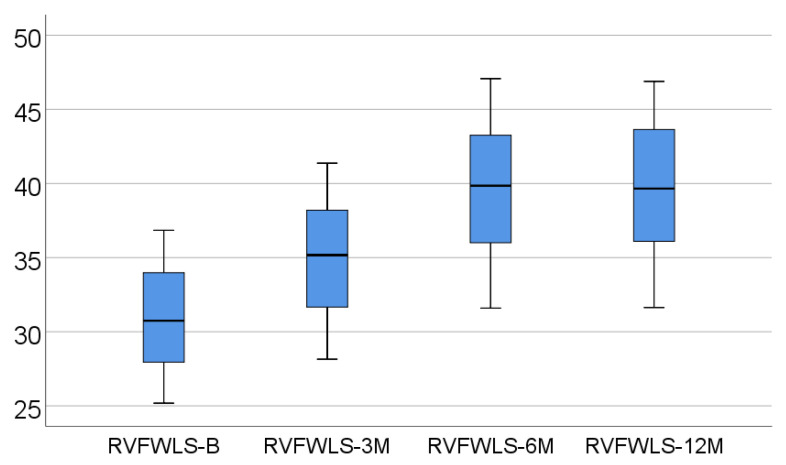
Box-plot representation of RVFWLS evolution.

**Figure 2:**
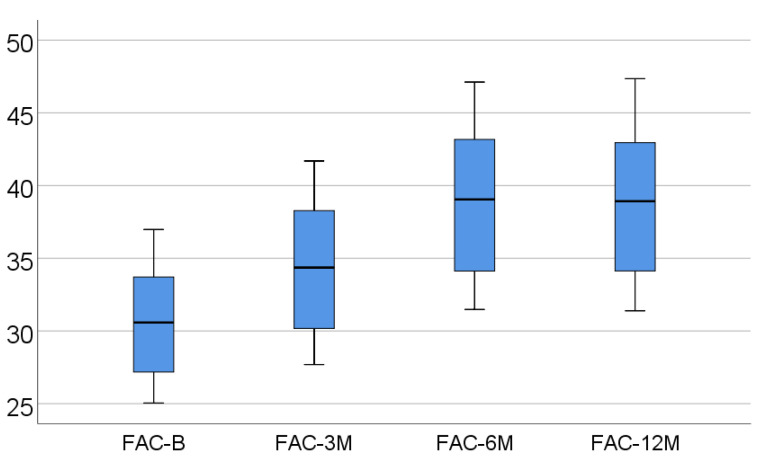
Box-plot representation of FAC evolution.

**Figure 3:**
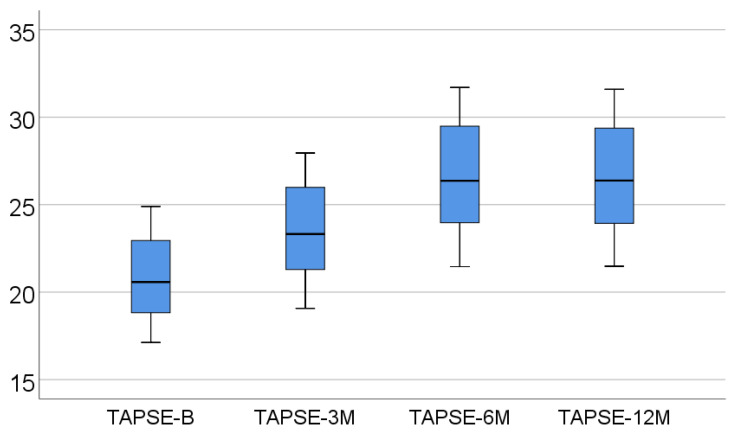
Box-plot representation of TAPSE evolution.

Data from Table 2 and Figure 1, Figure 2 and Figure 3 show the dynamic evolution of RVFWLS, FAC, and TAPSE values from baseline to 3/6/12 months. The distribution of parameters was assessed as non-parametric according to the Shapiro–Wilk test (*p* < 0.05). The differences between the parameters measured at different times were significant according to Friedman’s tests (*p* < 0.001), with RVFWLS, FAC, and TAPSE values having a significant increase at 3/6/12 months from baseline (*p* < 0.001) and a significant increase at 6 months vs. 3 months (*p* < 0.001). The differences between 12 months and 6 months, however, were not significant (*p* > 0.05), according to Dunn–Bonferroni post hoc tests.

**Figure 4:**
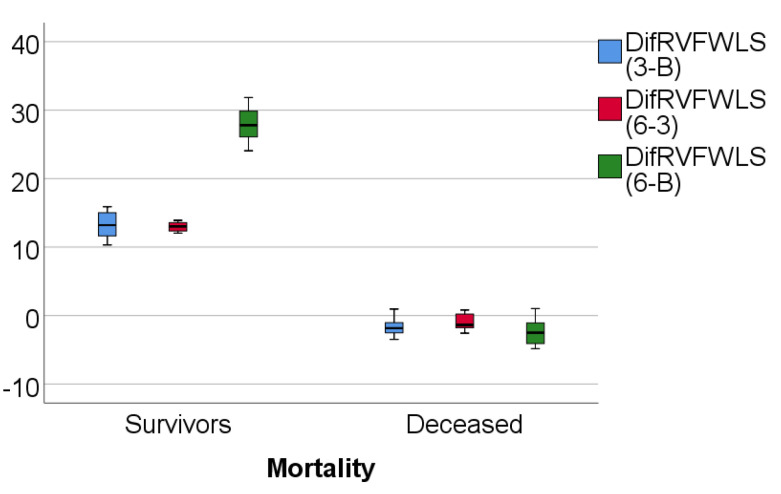
Box-plot representation of RVFWLS evolution in comparison with mortality.

**Figure 5:**
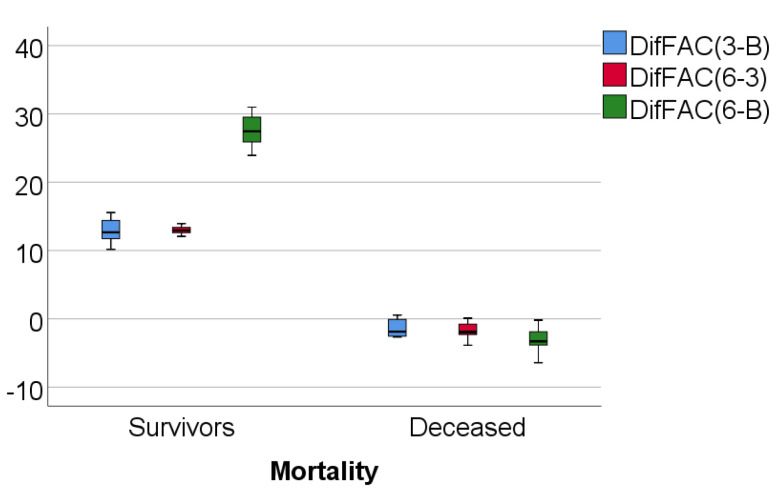
Box-plot representation of FAC evolution in comparison with mortality.

**Figure 6:**
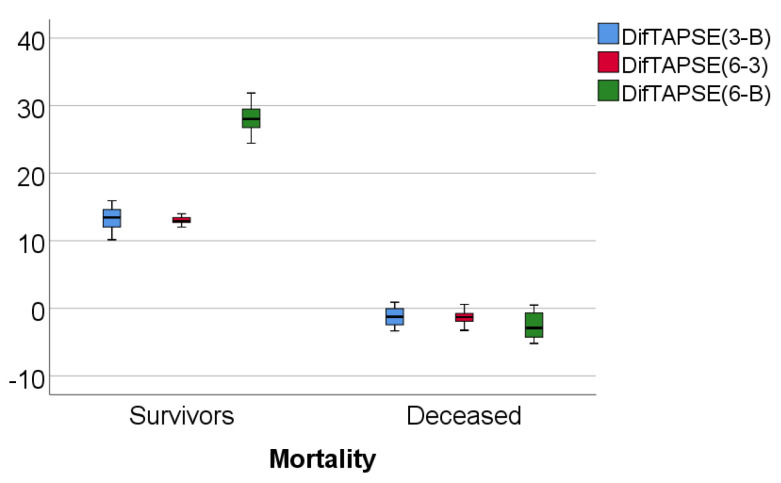
Box-plot representation of TAPSE evolution in comparison with mortality.

Data from Table 3 and Figure 4, Figure 5 and Figure 6 show RVFWLS, FAC, and TAPSE evolution values compared to mortality. The distribution of parameters was assessed as nonparametric according to the Shapiro–Wilk test (*p* < 0.05)**.** The differences observed of the evolution values in comparison to mortality were statistically significant according to Mann–Whitney U tests (*p* < 0.001), showing that the RVFWLS, FAC, and TASPE values had a higher increase at 3 months from baseline, 6 months vs. 3 months, and 6 months from baseline in survivor patients than in deceased patients (where these parameters had a decrease over time).

**Figure 7:**
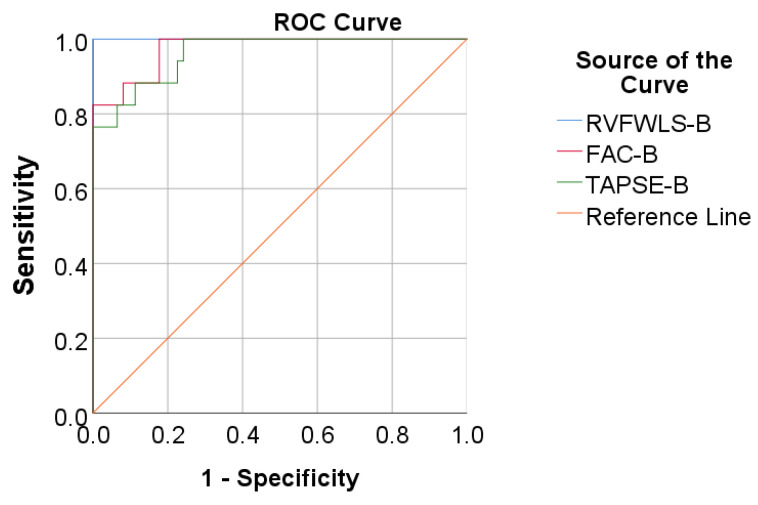
ROC curve using RVFWLS, FAC, and TAPSE baseline values for the prediction of mortality.

Data from Table 4 and Figure 7 show the ROC curve using RVFWLS, FAC, and TAPSE baseline values to predict mortality. All the studied parameters had a significant prediction over mortality (*p* < 0.001), having outstanding performance.

Using the ROC curve, cutoff values were established for each parameter:

- A baseline value lower than 18.5 for RVFWLS has a sensitivity of 94.1% and specificity of 100% for mortality prediction;
- a baseline value lower than 25.62 for FAC has a sensitivity of 88.2% and specificity of 90.1% for mortality prediction;
- a baseline value lower than 18 for TAPSE has a sensitivity of 88.2% and specificity of 85.5% for mortality prediction.

## 4. Discussion

This paper aimed to investigate whether the initiation of HD improves the RV function as judged by conventional methods (TAPSE or FAC) and by modern RV strain assessment using 2D-STE. Moreover, we focused on identifying the RV systolic function parameters that can hold a predictive value for one-year mortality.

Our research brings new evidence in a context of deep uncertainties reported by the international guidelines regarding the cardiovascular risks and benefits of dialysis initiation. There is a general conception that the first few months on dialysis are prone to high cardiovascular risks, though it is unclear whether the adverse events are triggered by dialysis initiation [11,12]. Heart failure (HF) during dialysis treatment is associated with high mortality [13].

High uncertainties derive from a few studies with extremely heterogeneous results that evaluated longitudinal changes in subclinical HF after initiation of dialysis: the CRIC study reported a decline in LVEF [14], the CASCADE [15] and IDEAL [16] trials showed no change in LVEF, and another study [17] reported improvement in LV parameters after HD initiation. These inconsistencies may support the idea of reverse causality, as those with higher eGFR values that commence dialysis may rather be at higher health risks because of frailty and accumulated comorbidities than because of the HD initiation itself [18].

As a consequence of these important inconsistencies, Kidney Disease: Improving Global Outcomes (KDIGO) guidelines encourage further clinical trials in which patients are followed beyond dialysis initiation [18]. Therefore, our paper contributes with new, valuable evidence to fill the scientific gap of post-HD cardiovascular monitoring.

RV function appears to play a central role in post-HD cardiovascular deterioration. A regression analysis [13] showed that arteriovenous fistula-induced RV dysfunction may contribute to LV dysfunction in dialysis patients, playing an essential role in triggering LV dysfunction through right-to-left ventricular interdependence. Conversely, LV dysfunction did not significantly influence RV parameters [13]. Therefore, our focus has fallen primarily on the assessment of post-HD RV systolic function and mechanics.

Our research has three main strengths. Chiefly, it is the first clinical study to assess long-term RV function after HD initiation. Secondly, it proposes a novel, modern alternative to evaluate RV function by RV strain assessment using 2D-STE. Thirdly, our results emphasize that a low RV strain has an important predictive power for mortality, irrespective of HD, suggesting that the high health risks associated with HD initiation may not be directly linked through a cause–effect mechanism.

In our prospective approach, there was a significant increase in RVFWLS, FAC, and TAPSE values at 3/6/12 months from baseline (*p* < 0.001) and, very interestingly, a statistically compelling increment was also observed at 6 months for all parameters mentioned (when compared to the 3 month analysis, *p* < 0.001). At the end of the study, RVFWLS, FAC, and TAPSE did not suffer any other variations compared to the 6 month evaluation (*p* > 0.05).

In the recent literature, there are inconsistent results regarding the sensitivity of TAPSE and FAC assessment on preload reduction by initiating HD. However, all these were short-term studies evaluating TAPSE or FAC within hours or days, but not months after HD initiation [19,20,21,22].

Although strain is considered independent of loading conditions, some studies reported a bettering in strain values ensuing elective HD within minutes or days [23]. We believe that the amelioration seen in all studied parameters at 6 months after HD initiation is a milestone in the life of an ESKD patient.

Hemodialysis can improve myocardial perfusion with a steady reduction amid the interdialytic hiatus [24,25]. In time this reduction could lower the cardiac chamber size or the pulmonary circulation loading [26], ultimately improving all the RV systolic indices, as confirmed by our outcomes.

Furthermore, concerning mortality, RVFWLS, FAC, and TAPSE values significantly decreased at 3 and 6 months in all 17 deceased patients.

We identified the fact that a baseline FAC less than 25.62% has a sensitivity of 88.2% and specificity of 90.1% for the prediction of mortality, and a baseline TAPSE less than 18 mm has a sensitivity of 88.2% and specificity of 85.5% for the prediction of mortality, our outcomes being similar with those already validated in more extensive studies and different population groups [8].

Nevertheless, these indicators may bear limitations for different reasons, such as patients with worse image quality, ventricular loading conditions, or even overall heart motion [27].

In contrast, speckle-tracking echocardiography grants appraisal of the myocardial mechanics in an angle-independent fashion. Our study depicts the fact that if a baseline RVFWLS value is less than 18.5%, this carries a sensitivity of 94.1% and specificity of 100% for the prediction of mortality at 6 or 12 months, an outcome that is similar with other studies, although different in populations or results whatsoever [28,29]. As stated above, this issue may suggest that a low value of RV strain carries the burden of mortality, irrespective of HD or any other aggravating or ameliorating factor.

This study has at least three limitations. Firstly, there is a small number of patients included, which reduces the generalizability of our conclusions. Secondly, our study is not comparative and randomized. Thirdly, there is a possibility of selection bias due to high mortality rates in our cohort.

## 5. Conclusions

Our study is the first to describe and investigate RV function by 2D-STE and correlate it with conventional echocardiography methods in patients with ESKD just before and after the initiation of HD. RV function was significantly ameliorated at 3/6 and 12 months compared to the baseline values (pre-HD). We found that TAPSE, FAC, and RVFWLS have an outstanding prognostic role concerning one-year mortality in this frail population.

## Figures and Tables

**Table 1:** Characteristics of the analyzed patients.

Parameter (*N* = 79)	Value
Age (Average ± SD, Median (IQR), Min–Max)	59.13 ± 15.41, 61 (52–71), 19–83
Gender (Nr., %)	35 (44.3%) F, 44 (55.7%) M
BMI (Average ± SD, Median (IQR), Min–Max)	26.71 ± 5.37, 26.67 (21.96–30.1), 16.44–42.5
Obesity (Nr., %)	59 (74.7%) −, 20 (25.3%) +
Smoking (Nr., %)	68 (86.1%) −, 11 (13.9%) +
HT (Nr., %) (Grades)	3 (3.8%) −, 2 (2.5%) I, 2 (2.5%) II, 72 (91.1%) +
Heart failure—NYHA Classification (Nr., %)	42 (53.2%) I, 34 (43%) II, 3 (3.8%) III
Diabetes mellitus (Nr., %)	53 (67.1%) −, 26 (32.9%) +
History of MI (Nr., %)	71 (89.9%) −, 8 (10.1%) +
CABG/PTCA (Nr., %)	76 (96.2%) −, 3 (3.8%) +
Hb (Average ± SD, Median (IQR), Min–Max) (g/dL)	9.75 ± 1.77, 9.7 (8.5–10.9), 5.7–14.6
Anemia (Nr., %)	50 (63.3%) −, 29 (36.7%) +
Uric acid (Average ± SD, Median (IQR), Min–Max) (mg/dL) (*N* = 70)	7.46 ± 1.92, 7.25 (6.1–9.025), 3.9–12.3
Hyperuricemia (Nr., %) (*N* = 70)	42 (60%) −, 28 (40%) +
EF% (Average ± SD, Median (IQR), Min–Max)	54.35 ± 9.74, 55(50–61), 23–70
EF% <40% (Nr., %)	74 (93.7%) −, 5 (6.3%) +
Mortality (Nr., %)	17 (21.5%)

IQR: Interquartile range; Nr: Number; BMI: Body Mass Index; HT: Hypertension; NYHA: New York Heart Association; MI: Myocardial Infarction; CABG: Coronary Artery Bypass Grafting; PTCA: Percutaneous Transluminal Coronary Angioplasty; EF: Left Ventricular Ejection Fraction.

**Table 2:** Dynamic evolution of RVFWLS, FAC, and TAPSE values from baseline to 3/6/12 months.

Parameter/Value (*N* = 62)		Baseline	3 Months	6 Months	12 Months	*p* *
RVFWLS	Average ± SD	31.06 ± 3.5	35.17 ± 4.03	39.72 ± 4.53	39.72 ± 4.58	<0.001
	Median (IQR)	30.7 (27.9–34)	35.1 (31.6–38.4) †	39.8 (35.8–43.4) †,**	39.6 (36–43.6) †,‡	
FAC	Average ± SD	30.7 ± 3.8	34.7 ± 4.38	39.2 ± 4.96	39.13 ± 4.96	<0.001
	Median (IQR)	30.5 (27.1–33.7)	34.3 (30.1–38.4) †	39 (34–43.4) †,**	38.9 (34.1–43.1) †,‡	
TAPSE	Average ± SD	20.8 ± 2.34	23.57 ± 2.71	26.63 ± 3	26.62 ± 3	<0.001
	Median (IQR)	20.5 (18.7–22.9)	23.3 (21.1–26) †	26.3 (23.9–29.5) †,**	26.3 (23.9–29.3) †,‡	

* Related-samples Friedman’s two-way analysis of variance by ranks, † (*p* < 0.001—in comparison to baseline values), ‡ (*p* > 0.05—12 month values vs. 6 month values), ** (*p* < 0.001—3 month values vs. 6 month values). RVFWLS: RV free wall longitudinal strain; FAC: fractional area change; TAPSE: tricuspid annular plane systolic excursion.

**Table 3:** Comparison of RVFWLS, FAC, and TAPSE evolution values in comparison to mortality.

Parameter/Value/Mortality (*N* = 71)			3 Months–Baseline	6 Months–3 Months	6 Months–Baseline
RVFWLS (%)	Average ± SD	−	13.21 ± 1.8	12.96 ± 0.61	27.89 ± 2.11
	Median (IQR)	−	13.2 (11.6–15.01)	13 (12.34–13.58)	27.8 (26.08–29.84)
	Average ± SD	+	−1.53 ± 1.56	−0.93 ± 1.24	−2.45 ± 2.1
	Median (IQR)	+	−1.84 (−2.82–−0.11)	−1.36 (−1.92–0.5)	−2.5 (−4.45–−0.65)
	*p* *		<0.001	<0.001	<0.001
FAC (%)	Average ± SD	−	12.93 ± 1.62	12.98 ± 0.5	27.6 ± 1.93
	Median (IQR)	-	12.65 (11.72–14.41)	12.94 (12.61–13.38)	27.45 (25.88–29.52)
	Average ± SD	+	−1.44 ± 1.26	−1.66 ± 1.24	−3.08 ± 2
	Median (IQR)	+	−1.87 (−2.6–−0.02)	−1.93 (−2.42–−0.5)	−3.29 (−4.48–−1.35)
	*p* *		<0.001	<0.001	<0.001
TAPSE (%)	Average ± SD	-	13.33 ± 1.52	13 ± 0.52	28.05 ± 1.82
	Median (IQR)	-	13.45 (12.02–14.66)	12.91 (12.7–13.42)	28.02 (26.75–29.5)
	Average ± SD	+	−1.22 ± 1.61	−1.4 ± 1.1	−2.6 ± 2.11
	Median (IQR)	+	−1.25 (−2.74–0.28)	−1.3 (−2.16–−0.7)	−2.91 (−4.37–−0.28)
	*p* *		<0.001	<0.001	<0.001

* Mann–Whitney U test.

**Table 4:** ROC curve using RVFWLS, FAC, and TAPSE baseline values for the prediction of mortality.

Parameter	Std. Error	*p*	Area (95% C.I.)
Baseline RVFWLS	0.000	<0.001	1.000 (1.000–1.000)
Baseline FAC	0.016	<0.001	0.974(0.942–1.000)
Baseline TAPSE	0.021	<0.001	0.962(0.920–1.000)

## Data Availability

Data used to support the findings of this study are available from the corresponding author upon request.
